# Pulpless Teeth, Roots, and Their Treatment

**Published:** 1896-10

**Authors:** Adam Flickinger

**Affiliations:** St. Louis.


ARTICLE IX.

### PULPLESS TEETH, ROOTS, AND THEIR TREATMENT.

BY ADAM FLICKINGER, D. D. S., ST. LOUIS.

Probably no single topic of the busy dental practitioner of to-day is receiving such earnest and thoughtful consideration as the treatment of pulpless teeth. For the past few years, even a casual reader of our numerous dental periodicals cannot have failed to observe the large amount of space devoted to treatises upon this important theme; which has been discussed from time to time by the dental societies throughout the land.

Roots, with proper therapeutic treatment, may be made to give service for years, in retaining the natural expression and contour of the face, by being crowned or used as piers for bridge-work.

This latter class of work, when properly constructed, is undoubtedly the most artistic and valuable substitute yet devised. For natural appearance, comfort, and stability it is unapproached by any other system of artificial teeth; especially so, if constructed on a plan which allows its easy removal for examination and the addition of such other work as may become necessary.

A proper preparation of the roots and teeth is, however, imperative, for upon this the future success of this class of work depends, and its importance cannot be overestimated.

In referring to the treatment of teeth and roots to be used for abutments and piers, or for crowning, I shall presume that we understand both those whose pulps have died (through some of the various causes) becoming putrescent, and those whose pulps have become exposed and must needs be devitalized. While I may not present a system entirely new in the treatment of the former, the method of its application is certainly not old, and if carried out carefully in detail it will be successful in the majority of cases, causing less annoyance to both patient and operator, by recurring inflammation, ulceration, etc., than any other method I have practiced.

The first step in the treatment of teeth in this condition is to gain free access into the pulp-chamber and canal or canals; then, in order that I may not force any of the contents through the apical foramen (causing additional trouble frequently harder to combat than the original one), I remove the contents carefully, using a nerve instrument, "home-made for the purpose," washing with warm water, and the sulfuric acid treatment so often described. In cases of tortuous canals much can be accomplished by sealing into the canals a ten per cent. solution of the acid for a day or so.

Having obtained a free and unobstructed passage, I apply the dam or other means of keeping the tooth perfectly dry, proceeding, if any abscess exists, with the beechwood creosote treatment, pumping it up and into the canal until it makes its appearance through the fistulous opening, after which I apply an electric wire attached to the alternating current and controlled by Holekamp-Moore, Grady & Co.'s alternating current controller, gradually increasing the heat in the ratio of thirty-four degrees to the minute. I then proceed with thymol or cassia, preferring the former for the anterior teeth, and apply the current again and again until every particle of oil is absorbed by the tooth.

When applying the current to the oil you will observe minute electrical sparks flying in all directions; the heat alone should be sufficient to destroy microbes, bacteria or any other kind of germs present.

I then fill the upper or apical part of the root with the following preparation, in the form of a paste composed of alum, thymol, glycerole, oxide zinc. Placing a small piece on an old broach with barbs removed, I work it up, not being over-particular how far; then take a small piece of heated gutta-percha, force it into the canal, which drives the preparation ahead, thoroughly filling the canal and sealing it better than any other preparation with which I am familiar, not excepting chloro-percha, oxyphosphate, wax, paraffin, etc.

For teeth and roots containing putrescent pulps, with no external opening or abscess, I use the same treatment, discarding, however, the creosote or carbolic acid, and proceeding with sulfuric acid, oils and electric wire.

This method I have adopted in all cases, have used it in all conditions and stages of inflammation and ulceration, in acute and chronic cases. I have treated and filled root and tooth immediately where a slight periosteal inflammation and swelling of the face still existed. In one case the tooth was so sore to the touch that it was impossible to use the mallet, yet I filled the root as described, and the tooth with gold, by hand-pressure; and to the great satisfaction of both myself and my patient the swelling and pain subsided after twenty-four hours, and to this day the tooth is doing well.

I may be accused of having gone to extremes, but so far the results have been so favorable that I feel the ends have justified the means.

In regard to mummifying pulps with the preparation of alum, thymol, glycerol, and oxid zinc, and acting upon the suggestion of Dr. Soderberg to "observe, compare, reflect, and record," I may state for the benefit of others that it is possible to mummify a pulp. I have experience for years, and have succeeded in a great many cases with a preparation of arsenious acid, menthol, thymol, and glycerol. In 1877 I treated three first permanent molars with this preparation for my wife. After nine years it became necessary to crown two of them. Without disturbing the former treatment, I placed gold shell crowns over them, and to this day the three teeth have never given any trouble whatever.

Since the publication of Dr. Soderberg's article, I have concluded to use the preparation in the proportion he suggests for the purpose of pulp-mummification, but hesitate to go to the extreme which he recommends, viz., to remove the contents of the pulp-chamber *only*, leaving the canals untouched. I have observed in my many experiments during the past twenty-three years with preparations too numerous to mention, that it is advisable to remove as much of the pulp as can easily be reached, though not necessarily all, mummifying the remainder. In fact, I think the day is not far distant when it will be an accepted fact that the successful treatment of devitalized teeth does not depend upon the complete removal of the pulp and the filling of roots to the apex, as is claimed to be imperative.

Science in general is making wonderful progress; why should we not solve this problem ?—*Dental Cosmos.*

